# Operative Factors Associated with Severe Hypotension in the Postoperative Setting in Patients with Carotid Artery Endarterectomy

**DOI:** 10.3390/life14111435

**Published:** 2024-11-06

**Authors:** Mircea Robu, Irina-Maria Margarint, Ovidiu Stiru, Andreea Raluca Hanganu, Bogdan Radulescu, Vlad Ichim, Gabriel-Petre Gorecki, Miruna Guzu, Claudia Mazilu, Vlad Anton Iliescu, Horatiu Moldovan

**Affiliations:** 1 Faculty of Medicine, Carol Davila University of Medicine and Pharmacy, 050474 Bucharest, Romania; mircea.robu@drd.umfcd.ro (M.R.); andreea.florea@drd.umfcd.ro (A.R.H.); bogdan.radulescu@umfcd.ro (B.R.); vladanton.iliescu@gmail.com (V.A.I.); horatiu.moldovan@umfcd.ro (H.M.); 2 Prof. Dr. C.C. Iliescu Emergency Institute for Cardiovascular Diseases, 022322 Bucharest, Romania; guzu.miruna@gmail.com (M.G.); dr.claudiamazilu@gmail.com (C.M.); 3 Department of Cardiovascular Surgery, Emergency Clinical Hospital Bucharest, 014461 Bucharest, Romania; ichim_vlad@yahoo.com; 4 Faculty of Medicine, Titu Maiorescu University, 031593 Bucharest, Romania; gabriel.gorecki@prof.utm.ro; 5 Academy of Romanian Scientists, 50711 Bucharest, Romania

**Keywords:** carotid endarterectomy, temporary shunting, stroke, hypotension

## Abstract

Background: Carotid endarterectomy is a recognized method for preventing stroke for both symptomatic and asymptomatic hemodynamically carotid artery stenosis. Hemodynamic depression is more frequently associated with carotid artery stenting, while postoperative hypertension and cerebral hyperperfusion syndrome are known frequent complications after carotid endarterectomy. Severe hypotension after carotid revascularization is associated with a higher risk of perioperative stroke, myocardial infarction, and death, with limited data existing regarding risk factors. This study aims to investigate intraoperative risk factors for severe hypotension after carotid endarterectomy. Methods: A total of 160 patients who underwent carotid endarterectomy were included in this study. Patients with other cardiac conditions that required concomitant cardiac surgery, patients with incomplete medical records, and patients considered high risk for surgery were excluded. Results: The incidence of severe hypotension was 30.6%. Patients with severe hypotension after carotid endarterectomy had a higher incidence of diabetes, moderate mitral valve regurgitation, a history of percutaneous coronary intervention, and higher operative times. Using logistic regression, temporary shunt insertion was independently associated with severe postoperative hypotension (OR = 2.26, 95% CI = 1.09–4.71, *p* = 0.029). Conclusions: This result favors the selective shunting strategy when performing carotid endarterectomies, especially for those patients with comorbidities predisposing to postoperative complications.

## 1. Introduction

Atherosclerotic extracranial cerebrovascular disease is a major cause of stroke and a leading cause of death [1]. Carotid endarterectomy (CE) was first reported by Eastcott in 1954 in a 66-year-old patient with recurrent transitory ischemic attacks (TIAs) [2]. It was the first time a surgical technique was considered as a treatment for a neurological condition such as cerebral ischemia [3]. The beneficial effect of this surgical technique was confirmed by the North American Symptomatic Carotid Endarterectomy Trial (NASCET) [4] and the European Carotid Surgery Trial (ECST) [5], especially in severe symptomatic carotid artery stenosis, both studies reporting similar rates of periprocedural stroke and in-hospital death.

Guidelines for the treatment of carotid artery disease were elaborated by the American Heart Association [6], the European Society for Vascular Surgery [7], and the European Stroke Organization [8]. CE is indicated in symptomatic (ipsilateral TIA or stroke within the past 6 months) with a 50% to 99% carotid artery stenosis, provided that the perioperative morbidity and mortality risk is low. All the society guidelines recommend that CE should be performed within 14 days from the onset of symptoms to increase the likelihood of a stroke-free outcome. For the asymptomatic 60% to 99% carotid artery stenosis, CE is indicated if there are one or more imaging or clinical signs associated with an increased risk of stroke.

Carotid revascularization is frequently complicated by hemodynamic complications [9]. Hemodynamic depression (HD) (bradycardia, hypotension, or both) occurs more often when carotid artery stenting is performed [10,11,12], while postoperative hypertension and cerebral hyperperfusion syndrome are known frequent complications after CE [13]. The incidence of HD is as high as 54.4% in CAS [14].

Severe hypotension is the consequence of transient baroreceptor dysfunction and carotid sinus manipulation [15,16] and is associated with a higher risk of perioperative stroke, myocardial infarction, and death after carotid artery surgery [14,17]. However, none of the traditional risk indexes encompasses the risk of HD and in particular the risk of severe hypotension [18], especially after CE.

Limited data are available regarding risk factors for severe hypotension after CE, in contrast with postprocedural hypotension in patients with coronary artery stenting (CAS). This study investigates the incidence of severe hypotension post-CE and the risk factors for this complication.

## 2. Methods

Between January 2020 and December 2023, 172 patients were admitted to our center, proposed for CE. Clinical characteristics and demographic data were collected from medical records and the electronic health system. Local ethics committees approved the protocol, and written informed consent was obtained from each patient.

Indication for surgery was performed after the European Stroke Organization guidelines and after neurological consultation.

The inclusion criteria included patients with symptomatic (ischemic stroke or TIA within 6 months) 50% to 99% carotid artery stenosis or patients with asymptomatic 70% to 99% carotid artery stenosis, assessed by carotid artery angiography or by contrast computed tomography of the cervical region.

The exclusion criteria included (1) patients with severe valvopathies (mitral/aortic or tricuspid valve) that required concomitant surgery; (2) patients with incomplete medical records; (3) patients considered high risk for surgery (symptomatic coronary artery disease, severe coronary artery stenosis diagnosed in the same setting at coronary angiography, NYHA III or IV heart failure); (4) patients with reduced life expectancy due to severe non-cardiac comorbidities like neoplasia; and (5) patients with initial severe carotid artery stenosis based on Doppler Ultrasound, not confirmed by contrast computed tomography of the cervical region or carotid artery angiography.

All patients were evaluated by a cardiology physician before surgery. Patients with a history or risk factors for coronary artery disease, angina or angina equivalent, or signs of coronary artery disease on transthoracic echocardiography were scheduled for coronary angiography before surgery.

The diagnosis of carotid artery stenosis was performed by initial Doppler ultrasound and confirmed by contrast computed tomography of the cervical region or carotid artery angiography.

Stroke was defined as an acute onset neurological deficit corresponding to an ischemic lesion on brain computed tomography or magnetic resonance imaging.

TIA was defined as a transient episode of neurologic dysfunction due to focal brain, spinal cord, or retinal ischemia without acute infarction or tissue injury according to 2009 AHA/ASA guidelines.

Severe hypotension was defined as mean arterial pressure <60 mmHg after volume resuscitation, requiring continuous vasopressor infusion (norepinephrine) in the first 12 h.

### 2.1. Surgical Technique

Standard and invasive monitoring including continuous blood pressure, central venous pressure, and urine output monitoring were employed through the perioperative setting. After the institution of general anesthesia, the patient is positioned on the operating table with the head extended and rotated to the opposite side of the carotid artery that is to be operated on. Near-infrared spectroscopy (NIRS) is used to monitor cerebral oxygenation. The common, external, and internal arteries are dissected and isolated with arterial loops, via a 10 cm longitudinal incision at the medial border of the sternocleidomastoid muscle. Intravenous heparin is administered before arterial clamping (50 IU/kg, maximum dose of 5000 IU). A longitudinal arteriotomy is made, beginning from the common carotid artery over the carotid bifurcation and extending distally to the internal carotid artery at the level of the normal arterial wall. We usually place a shunt in the following situations: (1) internal carotid artery (ICA) backflow unsatisfactory; (2) retrograde pressure at the level of ICA below 40 mmHg; and (3) a more than 15% drop in NIRS. Continuous transcranial Doppler ultrasonography and EEG monitoring are not available in our institution. Usually, plaque is removed down to the level of the intima, and two 7.0 polypropylene sutures are used to fix the layers at the distal end of the endarterectomy. The endarterectomy is completed using one of the following techniques: direct suture of the artery or a collagen and silver-coated polyester patch, or a saphenous vein patch sutured with a continuous 6.0 polypropylene suture. After completion of the endarterectomy, the temporary shunt is extracted, the arteries are de-aired and declamped, and patency is verified using intraoperative Doppler ultrasound. A surgical drain is inserted via contra incision, hemostasis is achieved, and the surgical wound is closed by suturing the anatomical layers.

### 2.2. Statistical Analysis

Wizard 2 Statistical Software for Mac OS (Wizard–Statistics & Analysis., Raipur, Chattisgarh, India) was used for statistical analysis. Summary statistics are presented as absolute numbers and percentages for categorical values and as the mean and standard deviation for continuous values.

The incidence of severe postoperative hypotension was investigated.

In order to investigate the association between preoperative and postoperative factors and severe postoperative hypotension, a multivariable analysis was performed using logistic regression, taking into account a model that included variables achieving a *p*-value < 0.1 in a univariate analysis. A predictive modeling strategy with the backward stepwise method of entering data was then used. Logistic regression results are presented as odds ratios (ORs) with confidence limits and *p*-values. *p* < 0.05 was considered statistically significant. Variables included in the univariate analysis were the following: age, history of smoking, chronic kidney disease, lower leg ischemia, hypertension, male sex, hyperlipidemia, diabetes, aortic, mitral, or tricuspid valve moderate or severe stenosis or regurgitation, severe left ventricular dysfunction, myocardial hypokinesia or akinesia, direct carotid artery arteriography, saphenous or silver-coated polyester patch, dual antiplatelet therapy, temporary shunt insertion, maximum levels of cardiac enzymes, left and right non-significant stenosis, 50–69% stenosis, 70–99% stenosis, CA occlusion, preoperative coronary angiography, one/two or three coronary vessels disease, history of PCI, history of stroke or TIA, new postoperative neurological complications, mean duration of surgery, mean duration of intubation, and prolonged ventilation (more than 12 h).

An independent *t*-test or Chi-square test was used to compare the groups of patients with and without severe postoperative hypotension.

## 3. Results

### 3.1. Pre/Intra/Postoperative Characteristics of the Study Population

A total of 172 patients with CE were included in this observational study. Out of these, 12 patients were excluded (9 patients with incomplete medical records, and 3 patients with overestimated carotid artery stenosis based on initial Doppler ultrasound—2 patients had carotid artery angiography in the same setting as coronary angiography and had an initial 70% carotid artery stenosis assessed by DU under 50% and 1 patient had a suboclusive carotid artery stenosis not confirmed by contrast computed tomography of the cervical region).

The general characteristics of the study populations are listed in Table 1. The mean age was 67.64 ± 7.54 years. Almost all patients had hyperlipidemia (93.1%) and arterial hypertension (91.9%), and 63.7% of them were on dual antiplatelet therapy. A history of stroke was present in 52.5% of patients, while 13.2% had a history of transient ischemic attack. Preoperative coronary angiography was performed in 35.7%; 10% had previous coronary artery bypass grafting, 8.8% had a history of percutaneous coronary artery intervention, and three-vessel coronary artery disease was present in 15% of the patients.

Eight patients (5%) had left ICA occlusion, while six patients (3.8%) had right ICA occlusion. Carotid artery stenosis 70% to 99% was present in 55% of cases on the right side and in 57.2% on the left side. Stenosis between 50% and 69% had an incidence of 11.9% on the left ICA and 12.5% on the right ICA.

Temporary shunt placement was inserted in 50.6% of the cases. CE was most often completed with direct carotid artery arteriography (37.5%), a saphenous vein patch was used in 31.9%, and a silver-coated polyester patch in 30% of the cases. The mean duration of surgery was 3.2 ± 0.71 h.

New neurological complications were encountered in eight patients with delirium, with hypoglossal nerve injury being the most frequent complication (1.9%). One patient developed an ischemic stroke, and another patient developed vagus nerve injury.

Severe hypotension requiring vasopressor support had an incidence of 30.6%. Severe hypertension was seen in 31.9% of the cases.

The mean duration of intubation time was 5.46 ± 3.68 h, with 15.6% of patients requiring more than 12 h of intubation.

### 3.2. Characteristics of Patients with Severe Hypotension

Table 2 shows the comparison between patients with and without severe hypotension. Regarding preoperative characteristics, patients with severe postoperative hypotension had a higher incidence of diabetes (*p* = 0.002), moderate mitral valve regurgitation (*p* = 0.045), and a history of PCI (*p* = 0.024). Intraoperative temporary shunt placement had a higher incidence in patients with severe hypotension (*p* = 0.034), and patients with severe postoperative hypotension had longer operative times (*p* = 0.04).

### 3.3. Factors Related to Severe Hypotension

The results of the univariate analysis of variables associated with severe postoperative hypotension with a *p*-value < 0.1 are presented in Table 3. Diabetes (OR = 0.166, 95% CI = 0.06–0.048, *p* < 0.001) and history of PCI (OR = 6.22, 95% CI = 1.65–23.46, *p* = 0.007) were included in the final model after backward selection. Temporary shunt placement was the only intraoperative variable associated with severe hypotension in the univariable analysis (OR = 2.1, 95% CI 1.05–4.19, *p* = 0.035). After model adjustment, shunt placement was independently associated with severe postoperative hypotension (OR = 2.26, 95% CI = 1.09–4.71, *p* = 0.029).

## 4. Discussion

Stroke is the second most common cause of death worldwide. The annual incidence of stroke has increased noticeably in the last decades because of the increase in obesity [19], and it is estimated that 25% of adults will have a stroke in their lifetime [20]. Moreover, half of the surviving patients have severe disability and are a great burden on the public health system [21].

In recent years, stroke prevention measures especially in elderly patients decreased stroke-associated disability, with a decrease in the absolute number of severely disabled (modified Rankin Scale 4–5) stroke patients at discharge [22].

CE has a beneficial effect on stroke prevention in patients with internal carotid artery stenosis. Moreover, in patients with TIA or non-disabling stroke, it is recommended to perform CE within 2 weeks of the index event rather than delay surgery to increase the likelihood of stroke-free outcome [8].

HD such as bradycardia, asystole, and hypotension occur during interventions on the carotid artery, especially in the setting of CAS, and are associated with major adverse cardiovascular events [14].

The incidence of HD after interventions for carotid artery revascularization is variable in different studies, ranging from 7.2% to 70% [14,23]. This non-concordance is probably due to marked heterogeneity in the definitions of HD. Gupta et al. and Bussiere et al. analyzed absolute values (systolic blood pressure < 90 mmHg, and heart rate < 60 bpm) [24,25], Cayne et al. investigated relative values comparing pre- and postoperative parameters [26], while Diehm et al. only investigated the need for treatment [27]. In our study, the incidence of severe postoperative hypotension was 30.6%, defined as mean arterial pressure < 60 mmHg after volume resuscitation, requiring continuous vasopressor infusion.

Bognioti et al. investigated HD after 237 carotid surgeries and included in this category both CE and CAS with an incidence of 54.4% [14]. Both intraoperative and postoperative hypotension had an incidence of 50.2%, and this included both cases with and without vasopressor infusion. The authors found an independent association between hypotension requiring vasopressors and major adverse cardiovascular events. The association between HD and worse outcomes is supported by Park et al. [12] (myocardial infarction, length of stay, and death) and Ullery et al. (stroke) [17].

Limited data are available regarding incidence and risk factors for postoperative hypotensive after CE. Park et al. analyzed 1474 CE and 157 CAS patients and found an incidence of clinically significant hypotension of 12.6% [12], while Altinbas et al. found an incidence of 7.2% [16]. Bogniotti et al. found asymptomatic carotid stenosis, endovascular surgery, and intraoperative hypotension or bradycardia independent predictors for HD in 237 carotid surgeries, including CE and CAS [14]. While we did not investigate intraoperative hypotension or bradycardia, the incidence of asymptomatic carotid artery stenosis was 47.5% (76 patients), and there were no significant differences between patients with and without postoperative severe hypotension. It should be mentioned that, in the Bogniotti study, the hypotension group included both patients with systolic blood pressure below 90 mmHg or mean arterial pressure below 60 mmHg and also the cases requiring vasopressors. In our study, we only included patients with vasopressors, and the smaller number of cases could justify why we could not confirm Bogniotti’s findings. Park et al. found that preoperative nitrate use and a history of tobacco use were correlated with postprocedural hypotension in patients with CE and CAS [12]. Tobacco use had an incidence of 38.1% (61 patients) in our study, with no significant differences. It should be mentioned that because of the retrospective nature of this study, the timing and the magnitude of tobacco use could not be fully characterized, and this is a limitation of our study. Similarly, we did not consider investigating the periprocedural use of nitrates or any other medication because of the inability to fully determine medication compliance and timing before CE. Also, the management of different comorbid conditions is not possible with this type of study. Both studies included patients with CE and CAS, and this could also be a reason why we could not confirm some of the risk factors found for severe postoperative hypotension.

One of the few studies investigating risk factors for postprocedural hypotension in CE was performed by Gibbs et al. [28]. They did not find any demographic or procedural factors associated with postoperative hypotension, and they also concluded that hypotension after CE was transient and not associated with adverse outcomes.

In contrast, in patients with CAS, postprocedural hypotension is well studied, and multiple risk factors have been identified (Table 4).

Temporary shunt placement is a method used for preventing cerebral hypoperfusion during CE [40]. Currently, there are two strategies for shunt placement (routine shunting [41] and selective shunting [42]), with no definitive guidelines. Those who propose selective shunting rely on the fact that most perioperative complications are the result of thrombo-embolic episodes rather than cerebral hypoperfusion and also that mortality and morbidity do not differ significantly when comparing routine vs. selective shunting [43]. Complications associated with shunt insertion such as intimal injury with thrombosis and embolization, pseudoaneurysm, distal embolization at insertion, and rupture of the distal end of the internal carotid artery are well described and have serious adverse effects on the prognostic of the patient [44,45,46].

In our study, the strategy for shunt insertion was selective shunting in the following instances: contralateral carotid artery occlusion, ipsilateral stroke, significant reduction in transcutaneous cerebral oximetry measurement parameters, and inadequate backflow from the ICA after clamping. The incidence of shunt placement was 50.6% of patients. With this strategy, the rate of complication was the following: one patient developed postoperative ischemic stroke with a modified Rankin Scale of 3 at discharge, two patients had cranial nerve injuries, and three patients experienced postoperative delirium.

The association between shunt placement and severe postoperative hypotension has not been communicated so far.

A possible mechanism for this can be a longer duration of surgery because of the technical aspect associated with shunt insertion. Besides the complications listed above, which are time-consuming, the surgical field is reduced, especially the lumen of the internal carotid artery after plaque extraction. In this region, separate 7.0 polypropylene sutures are placed to fixate the remaining intima to the rest of the arterial wall. Placement of these sutures is more time-consuming in the setting of temporary shunting. Also, the entire process of plaque excision is more difficult because of the need to permanently mobilize the shunt for a better visualization of the carotid lumen. Shunt extraction can also be time-consuming and technically difficult, especially when a patch is used to finalize the CE. Loosening the suture used to secure the patch can happen; additionally, sutures are required to achieve hemostasis, prolonging the surgery time. Also, it is more challenging and time-consuming to suture a patch at the native carotid artery wall with a shunt in place.

We investigated intraoperative risk factors for the development of severe postoperative hypotension such as temporary shunt placement, duration of surgery, suture of a patch, or direct arteriography. Unlike preoperative variables, intraoperative variables depend on the surgical team and can be modified. The aim is a surgical technique that can benefit the patient in the intensive care setting, reducing the risk of hemodynamic complications like severe hypotension.

This is an observational study and, as such, is vulnerable to the biases inherent to this type of analysis. Our sample size might still be considered small to have detected significant differences between those with and without severe postoperative hypotension. Further studies are needed to investigate intraoperative risk factors for hemodynamic complications after CE.

## 5. Conclusions

Data from 160 patients with CE were analyzed. Severe postoperative hypotension had an incidence of 30.6%, and patients with these complications had a higher incidence of diabetes, moderate mitral valve regurgitation, a history of PCI, and longer operative times. Temporary shunt placement was the only intraoperative factor associated with severe postoperative hypotension. This result can be explained by longer operating times because of the technical aspects associated with this method of cerebral protection. Considering this result, the selective shunting strategy could be more beneficial for the patient, especially for those with comorbidities predisposing to postoperative complications. Shunting could be considered only in specific situations such as contralateral subocclusion or occlusion, or severe stenosis on both vertebral arteries. However, further studies are needed to sustain this finding and clarify the need for shunting in CE.

## Figures and Tables

**Table 1:** General characteristics of patients.

	*n* = 160
Preoperative	
Age (years)	67.64 ± 7.54
Male sex	106 (66.2)
Smoking	61 (38.1)
Hyperlipidemia	149 (93.1)
Hypertension	147 (91.9)
Diabetes	46 (28.7)
Chronic kidney disease	20 (12.6)
Peripheral arterial disease	26 (16,2)
Dual antiplatelet therapy	102 (63.7)
History of stroke	84 (52.5)
History of TIA	21 (13.2)
Symptomatic patients	105 (65.62)
Severe left ventricular disfunction	3 (1.87)
Mitral valve annuloplasty	1 (0.6)
Moderate mitral valve regurgitation	8 (5)
Severe mitral valve regurgitation	1 (0.6)
Aortic valve prosthesis	2 (1.2)
Moderate aortic valve regurgitation	2 (1.2)
Moderate aortic valve stenosis	1 (0.6)
Left ventricle hypokinesia	21 (13.1)
Left ventricle akinesia	6 (3.8)
Preoperative coronary angiography	57 (35.7)
Non-significant coronary artery stenosis	13 (8.1)
One-vessel disease	14 (8.8)
Two-vessel disease	13 (8.1)
Three-vessel disease	24 (15)
History of CABG	16 (10)
History of PCI	14 (8.8)
Non-significant left ICA stenosis	26 (16.4)
50% to 69% left ICA stenosis	19 (11.9)
70% to 99% left ICA stenosis	91 (57.2)
Left ICA occlusion	8 (5)
Non-significant right ICA stenosis	35 (22)
50% to 69% right ICA stenosis	20 (12.5)
70% to 99% right ICA stenosis	88 (55)
Right ICA occlusion	6 (3.8)
Intraoperative	
Direct carotid arteriorrhaphy	60 (37.5)
Silver-coated polyester patch	48 (30)
Saphenous vein patch	51 (31.9)
Temporary shunt	81 (50.6)
Operative time (hours)	3.2 ± 0.71
Postoperative	
Neurological complication	8 (5)
Ischemic stroke	1 (0.6)
Delirium	3 (1.9)
Hypoglossal nerve injury	3 (1.9)
Vagus nerve injury	1 (0.6)
Hypertension	51 (31.9)
Bradycardia	14 (8.75)
Severe hypotension	49 (30.6)
Maximum CK (IU/L)	229.94 ± 464.39
Maximum CK-MB(IU/L)	24.26 ± 21.77
Duration of intubation (hours)	5.46 ± 3.68
Prolonged ventilation	25 (15.6)

TIA: transient ischemic attack; CABG: coronary artery bypass grafting; PCI: percutaneous coronary intervention; ICA: internal carotid artery; mean ± SD; n (%).

**Table 2:** Comparison between patients with and without severe hypotension.

	Severe hTA (+) *n* = 49	Severe hTA (−) *n* = 111	*p*
Age (years)	68.85 ± 7.62	67.1 ± 7.48	0.550
Male sex	36 (73.5)	70 (63.1)	0.199
Smoking	22 (44.9)	39 (35.1)	0.241
Hyperlipidemia	46 (93.9)	103 (92.8)	0.803
Hypertension	43 (87.8)	104 (93.7)	0.205
Diabetes	6 (12.2)	40 (36)	0.002
CKD	7 (14.6)	13 (11.7)	0.616
PAD	7 (14.3)	19 (17.1)	0.655
DAPT	31 (63.3)	71 (64)	0.932
Angina grade (CCS)			
CCS I	8 (16.3)	19 (17.1)	0.902
CCS II	6 (12.2)	13 (11.7)	0.923
CCS III	1 (2)	6 (5.4)	0.338
History of stroke	25 (51)	59 (53.2)	0.803
History of TIA	5 (10.4)	16 (14.4)	0.494
Severe LV disfunction	1 (2)	1 (0.9)	0.550
Valve dysfunction	6 (12.2)	7 (6.3)	0.274
Left ventricle hypokinesia	8 (16.7)	13 (11.7)	0.397
Left ventricle akinesia	3 (6.1)	3 (2.7)	0.294
Preoperative coronary angiography	13 (26.5)	44 (39.6)	0.110
Non-significant coronary artery stenosis	4 (8.2)	9 (8.1)	0.991
One-vessel disease	4 (8.3)	10 (9)	0.890
Two-vessel disease	3 (6.1)	10 (9)	0.538
Three-vessel disease	8 (16.3)	16 (14.4)	0.755
History of CABG	3 (6.1)	13 (11.7)	0.277
History of PCI	8 (16.3)	6 (5.4)	0.024
Non-significant left ICA stenosis	6 (12.5)	20 (18)	0.388
50% to 69% left ICA stenosis	4 (8.3)	15 (13.5)	0.355
70% to 99% left ICA stenosis	27 (56.2)	64 (57.7)	0.869
Left ICA occlusion	4 (8.3)	4 (3.6)	0.210
Non-significant right ICA stenosis	12 (25)	23 (20.7)	0.550
50% to 69% right ICA stenosis	9 (18.4)	11 (9.9)	0.136
70% to 99% right ICA stenosis	25 (51)	63 (56.8)	0.501
Right ICA occlusion	1 (2)	5 (4.5)	0.450
Intraoperative			
Direct carotid arteriorrhaphy	17 (34.7)	43 (38.7)	0.626
Silver-coated coated polyester patch	16 (32.7)	32 (28.8)	0.627
Saphenous vein patch	16 (32.7)	35 (31.5)	0.888
Temporary shunt	31 (63.3)	50 (45)	0.034
Operative times (hours)	5.33 ± 3.79	4.52 ± 3.64	0.04
Postoperative			
Neurological complication	2 (4.08)	6 (5.04)	0.918
Hypertension	15 (30.6)	36 (32.4)	0.820
Prolonged intubation	7 (14.3)	18 (16.2)	0.757
Maximum CK (IU/L)	153.51 ± 149.57	263.1 ± 546.78	0.533
Maximum CK-MB(IU/L)	23.67 ± 12.55	24.53 ± 24.89	0.618

CKD: chronic kidney disease, PAD: peripheral arterial disease; DAPT: dual antiplatelet therapy; TIA: transient ischemic attack; CABG: coronary artery bypass grafting; PCI: percutaneous coronary intervention; ICA: internal carotid artery; LV: left ventricle; mean ± SD; *n* (%).

**Table 3:** Factors associated with severe hypotension (multivariable statistics).

	OR	95% CI	*p*	OR	95% CI	*p*
Shunt	2.1	1.05–4.19	0.035	2.26	1.09–4.71	0.029
Diabetes	0.25	0.1–0.63	0.004	0.166	0.06–0.48	<0.01
History of PCI	3.42	1.12–10.45	0.031	6.22	1.65–23.46	0.007
Moderate mitral valve regurgitation	4.147	0.95–18.12	0.059			

PCI: percutaneous coronary intervention.

**Table 4:** Main studies investigating risk factors for postprocedural hypotension after CAS.

Author/Year	No. Patients/Procedure	Risk Factors for Postprocedural Hypotension
Taha et al./2008 [29]	132 CAS	Male sex, preprocedural major stroke, carotid bulb lesion, contralateral occlusion
Lin et al./2007 [30]	416 CAS	Age >78 years, ejection fraction <25%
Yang et al./2024 [31]	2843 CAS	Age >65 years, stenosis site (bulb), severe stenosis, stenosis proximity to bifurcation, calcified plaques, post-balloon dilatation, bilateral CAS, intravenous fluid intake/mL in the first postoperative day
Rubio et al./2019 [32]	90 CAS	Carotid bifurcation/bulb involvement, preoperative regimen of two or more antihypertensives
Choi et al./2021 [33]	128 CAS	Carotid bulb involvement of a stenotic lesion
Pappada et al./2006 [34]	51 CAS	Ratio between the pre- and post-stenting diameter of the internal carotid artery
Qureshi et al./1999 [35]	51 CAS	Intraprocedural hypotension, previous ipsilateral endarterectomy
Wu et al./2015 [36]	191 CAS	Octogenarians, angina, contralateral occlusion
Rhim et al./2016 [37]	66 CAS	Calcifications, eccentric stenosis, extensive plaque distribution
Nonaka et al./2005 [38]	31 CAS	Distance between carotid bifurcation and maximum stenotic lesion, type of stenosis, calcifications at bifurcation
Wei et al./2023 [39]	162 CAS	Hypertension, previous stroke/TIA, CAD, and distance from the carotid bifurcation to the lesion with maximum stenosis

## Data Availability

Data are available upon request.
